# The impact of frailty on oral care behavior of older people: a qualitative study

**DOI:** 10.1186/1472-6831-13-61

**Published:** 2013-11-01

**Authors:** Dominique Niesten, Krista van Mourik, Wil van der Sanden

**Affiliations:** 1 Department of Global Oral Health, College of Dental Sciences, Radboud University Nijmegen Medical Centre, PO Box 9101HB, Nijmegen, The Netherlands; 2 Department of Public Health and Primary Care, Leiden University Medical Centre, Leiden, The Netherlands

**Keywords:** Aged, Oral health, Frailty, Oral hygiene, Dental care, Dental health services, Self-worth, Health behavior, Toothbrushing

## Abstract

**Background:**

Frailty has been demonstrated to negatively influence dental service-use and oral self-care behavior of older people. The aim of this study was to explore how the type and level of frailty affect the dental service-use and oral self-care behavior of frail older people.

**Methods:**

We conducted a qualitative study through 51 open interviews with elders of varying frailty in the East-Netherlands, and used a thematic analysis to code transcripts, discussions and reviews of the attributes and meaning of the themes to the point of consensus among the researchers.

**Results:**

Three major themes and five sub-themes emerged from our analyses. The major themes indicate that frail elders: A) favor long-established oral hygiene routines to sustain a sense of self-worth; B) discontinue oral hygiene routines when burdened by severe health complaints, in particular chronic pain, low morale and low energy; and C) experience psychological and social barriers to oral health care when institutionalized. The subthemes associated with the discontinuation of oral care suggest that the elders accept more oral pain or discomfort because they: B1) lack belief in the results of dental visits and tooth cleaning; B2) trivialize oral health and oral care in the general context of their impaired health and old age; and B3) consciously use their sparse energy for priorities other than oral healthcare. Institutionalized elderly often discontinue oral care because of C1) disorientation and C2) inconveniencing social supports.

**Conclusion:**

The level and type of frailty influences people’s perspectives on oral health and related behaviors. Frail elders associate oral hygiene with self-worth, but readily abandon visits to a dentist unless they feel that a dentist can relieve specific problems. When interpreted according to the Motivational Theory of Life Span Development, discontinuation of oral care by frail elderly could be viewed as a manifestation of adaptive development. Simple measures aimed at recognizing indicators for poor oral care behavior, and providing appropriate information and support, are discussed.

## Background

There is abundant evidence of a discrepancy between perceived oral treatment need and dental service-use by older people, a discrepancy that has persisted for more than 35 years [1-6]. Studies have indicated that, of a group of non-institutionalized elderly people with clinically assessed or normative oral treatment needs, about half perceived the need and about one quarter sought treatment [4,7]. Recent studies among elderly residents in Dutch and Italian nursing homes showed even larger differences between normative and self-perceived needs [8,9]. Apparently, large discrepancies exist between self-perceived and normative treatment need, and between self-perceived treatment need and service-use.

Frailty, as a “dynamic state affecting an individual who experiences losses in one or more domains of human functioning (physical, psychological, social)” [10], is likely to contribute to these discrepancies by negatively affecting both dental service-use and oral hygiene-related behaviors [11]. However, despite extensive research on barriers to dental service-use [7,12-17] and oral hygiene-related behavior [18-21] in which barriers have been associated with impaired mobility, impaired activities of daily living, low energy, depression, and lack of social support, it remains unclear how frailty in its many forms influences the oral care of older people. For example, it is not clear whether service-use and toothbrushing are disturbed more by impaired mobility, dexterity, or low morale, or, as some [22,23] suggest, by a lack of time and energy caused by more pressing general health problems. Nor do we know what motivates frail people to apply oral care despite physical and cognitive impairments, or why there are discrepancies between perceived treatment need and service-use. This knowledge should help to make evidence-based decisions about the allocation of resources aimed at improving the oral health related quality of life of people who are affected by frailty.

This study aims to explain **how frailty influences dental service-use and oral self-care by older people.**

## Methods

Open-ended or in-depth interviews [24] (p.12) were conducted with a group of elderly participants selected purposively for maximum variation in response to the topic guide [25]. This strategy allowed us to identify common patterns in responses across people with maximum variation in variables that are known to influence the oral health behavior of the target group: age [22,26-28], gender [26,27], dental status [15,22,28,29], institutionalization [13,14] and type and intensity of care they receive as a measure for frailty [15,16,20,21,30,31].

Regular daycare centers and assisted-living homes in the Arnhem-Nijmegen region, East-Netherlands, were randomly chosen from a national website that lists all Dutch care institutes (http://www.zorgkaartnederland.nl/). Most of the care-managers we contacted, agreed to participate. We asked them to identify potential participants according to the type and intensity of care they receive, based on the classification used by the Dutch National Centre for Indication of Care Need (CIZ). Each resident is assigned a ‘Package of Care Dependency’ (ZZP), indicating the level and type of care needed and ranging from ZZP-1 (mild frailty) to ZZP-6 (severe frailty) (Table 1), and from ZZP-7 to ZZP-10. ZZP values are assigned by a medical authority We excluded residents with ZZP values 7–10 because their health status is beyond ‘frail’: They are either completely functionally dependent, cognitively disabled, or receive end-of-life care. The care-manager and the interviewer informed each participant about the study and the interview methods. According to the care-managers, most recruits consented to participate. Reasons for non-participation were not collected. All participants were 65 years or older and gave informed consent in writing with the approval of the Medical Ethics Committee (CMO) of the UMC Nijmegen (CMO ref. 2009/153).

**Table 1:** ZZP*-scores, physical status, disorders and intensity of care associated with the participants

**ZZP-score**	**Physical status**					**Disorders**		**Hours/week of care needed**
	**Social coping**	**Psychosocial functioning**	**Personal care**	**Mobility**	**Motor functioning**	**Medical care**	**Behavioural disorders**	
1	+	0	+	+	0	0	0	3-5
2	+++	+	++	+	+	+	0	5,5-7,5
3	++++	++	++++	+++	++	+	0	9,5-11,5
4	++++	+++	++	+	+	+	+	11-13,5
5	+++++	++++	++++	++++	++	+	+	16,5-20
6	++++	+++	+++++	+++++	+++	++	0	16,5-20

“0” - no care needed; “++” - coaching needed; “++++” – some support needed; “++++++” – full support needed. *Zorgzwaartepakketten (care level package) V&V Enschede 2010 PJ/10/1657/imz.

### Data collection and analysis

Two trained interviewers (DN, KM) conducted the open-ended interviews with 51 participants (Table 2) between 2009 and 2012. We used an interview guide to focus attention on: 1) self-reported oral and general health; 2) oral self-care; and 3) use of dental services. We made observational notes to record events that might have influenced our interpretation of the interviews. In most cases, and in every case where we received any unclear or contradictory information from the participant, we contacted care-managers after the interview, either in person or by telephone, in order to briefly discuss our interpretation of the information.

**Table 2:** Participant characteristics

**Participant characteristics**	**Number**
*Sex*	
Female	35
Male	16
*Age*	
65-80 yr	24
>80 yr	27
ZZP-score*	
ZZP1	14
ZZP2-3	17
ZZP4-6	20
*Dental status*	
Natural teeth only	15
Nat. teeth and partial dentures	12
Nat. teeth and full upper dentures	12
Full upper and lower dentures	12
*Institutionalized*	
Yes	28
No	23

*Zorgzwaartepakketten (care level package) V&V Enschede 2010 PJ/10/1657/imz.

Interviews occurred in a quiet room within each facility or centre or in the participant’s private room or home. Data were collected on the age, chronic disorders, use of dental prostheses (all self reported), and ZZP scores (medical record) of each participant. Substitutes for missing ZZP scores were derived through consultation with the care-manager. All interviews were audio-taped, transcribed verbatim, and the identity of each participant was masked to maintain anonymity.

In order to identify the specific themes relating to the care behavior of the participant [24] (p.67), DN and KM first applied line-by-line coding of each transcript. We then discussed and reviewed the attributes and meanings of the codes until consensus was reached. This way, a coding frame developed. The coding process and analysis was supported by a computer program (MaxQDA 2010; http://www.MaxQDA.com) which also facilitated (semi-) quantification of codes and emerging themes during the analysis. A third researcher (WS) checked the reliability of the attribution of codes in five randomly selected interviews. DN and KM grouped coded segments with related content into code groups. We then formulated an initial set of themes based on the underlying meaning of grouped coded segments. Themes were repeatedly compared with the data following a method of ‘constant comparison’ [24] (p.71). We applied this method after every two or three interviews in order for emergent themes to be verified and explored in interviews that followed. The discussion and subsequent refining of themes among all authors went on until we reached consensus on a definite set of themes.

The analysis included the identification of the specific influence of different levels of frailty (ZZP 1–6) on care behavior both within and between transcripts by the references to frailty or related conditions, such as impairments or disabilities. In order to increase comprehensibility in the reporting phase, we hereby distinguished between slight frailty (ZZP 1), moderate frailty (ZZP 2 and 3), and severe frailty (ZZP 4, 5, and 6).

### Reflexivity of the researchers

Insights from various academic and professional backgrounds influenced the data analysis. The researchers added expertise in and knowledge of public oral health care and philosophy (DN), health sociology and medical anthropology (KM), dentistry and dental care (WS), and qualitative methodology (DN and KM) to the analysis. The only dental professional of the team did not conduct the interviews in order to reduce the chance of participants feeling restricted in their responses.

During the study design and in the analysis phase, we repeatedly consulted geriatric dentists and geriatric nurses to help us to bring up relevant issues during the interviews, and to create more contextual background to understand the participant’s information.

### Qualitative rigor

Several techniques helped to ensure the trustworthiness and credibility of our analysis [32]. Firstly, we combined or triangulated information from three sources: interviews; observational notes; and the opinions of care-managers. Secondly, the research team brought three separate professional backgrounds to the analysis. Thirdly, the interviewers carried out member checks during the interviews, which involved restating or summarizing information and then asking the participants to determine the accuracy. Lastly, we offer direct quotes from the transcripts to support our thematic interpretations. We stopped interviewing when no new themes or subthemes emerged (theme saturation) [33].

## Results

The views and experiences on oral health behaviors of most slightly frail (ZZP 1) and some of the moderately frail (ZZP 2 and 3) participants were very similar. They said that their oral hygiene routines had not altered much since their youth or early adulthood. All brushed their teeth daily and nearly everyone visited a dentist regularly.

The effects of frailty on oral care behavior only clearly manifested themselves for about half of the moderately frail (ZZP 2–3) and most of the severely frail (ZZP 4–6) people. The themes presented below are therefore predominantly, but not exclusively, based on their accounts. Apart from frailty levels and frailty factors, we paid attention to the factors age, gender, dental status and being institutionalized in case these appeared to influence the theme.

We identified three main themes and six subthemes relating to oral care behaviors of frail people. Quotes that best illustrate these themes are provided in Tables 3, 4 and 5.

**Table 3:** Themes A and supporting quotations

***Theme A: Adhering to routines in order to sustain a sense of self worth***	
A1	*A while ago, I was in hospital for a week where they gave me a special bowl to brush my teeth in. I find that awful, very awful. But there’s no way around it when you can’t stand up. […] I still think I should not skip brushing. […] I wish to feel clean. (woman, 70, severely frail, severe Parkinson).*
A2	*I just wanted to feel normal again. When you do your daily routines, combing your hair, brushing your teeth, just like you always do, it feels as if you’re not that ill. (man, 75, talking about his recent stay at the intensive care unit after acute renal failure).*
A3	*I wish to be cared for, I don’t won’t to lie here as a pile of old dirt, that goes for the mouth, for everything. (woman, 86, severely frail).*
A4	*If a nurse talks to me and brushes my teeth and then she says, well that’s nice and fresh like this, by saying so she lets me know that I still count as a human being. (woman, 80, slightly frail).*
A5	*You owe it to yourself to maintain a healthy mouth […] I live healthily, I hardly ever take sweets and I brush my teeth every night. (woman, 94, moderately frail).*
A6	*I like to care for my teeth [….] I like to be able to care for my teeth. It is so important that you don’t neglect your personal care […] they have told me that I have always looked so well after my body and my teeth […] that makes me proud. (woman, 70, severely frail, severe Parkinson).*
A7	*In that case [if she would not brush and her teeth would be visibly unclean] I’m quite sure that people would think ‘can’t that person brush her own teeth anymore’? (woman, 78, moderately frail).*
A8	*I thought, all those nurses, they get quite close to you. […] I would really dislike it if they would see me as mister rotting. […] as someone who is too slack to prevent the decay that after all he can do something about. (man, 75, severely frail, talking about his recent stay in hospital).*

**Table 4:** Theme B and supporting quotations

***Theme B: Lack of motivation: the benefits of dental visits or daily tooth cleaning are not worth the effort***	
***Subtheme: lack of belief in results***	
B1	*It’s not that I don’t want to go, but whom should I see? From what I have come across, it is only misery. (man, 93, full dentures, severely frail).*
B2	*When I take my dentures out, it feels freed. But I have to wear them, so… You think what could be done about it, I understand, but if I would have believed that a dentist could help me, I would have gone there a long time ago. But I know that it wouldn’t help. (woman,86, full dentures, slightly frail).*
B3	*I’ve got this feeling that my lower jaw is shrinking a lot. There’s hardly anything left there. But that’s a family thing, my mum had that too. (woman, 85, full dentures, moderately frail).*
B4	*They [dentures] have not been sitting well from the beginning. But I’ve always thought that it was because of this fungal infection, I had in my gullet. [..] That that infection has moved up to my mouth. […] Cause my mum had the same, her mouth was always sore. […] And her gums were sore too. And then she was rubbing like this. […] I have determined for myself that it really is that fungal infection. […]. And I won’t go to the dentist, because that is no use, they cannot fix it. (woman,86, full dentures, slightly frail).*
B5	*I don’t go anymore. He [a dentist] can’t do anything for me, can he? […] Last time I went was 10 years ago, and ever since I have not had any complaints, so why should I go? (woman, 85, dentate, moderately frail).*
B6	*Well I have tried to clean them [dentures] with a brush, but they weren’t that dirty, and they didn’t get that clean either […] well, no moss grows on them [dentures], what else should you care about? (man, 93, full dentures, severely frail).*
***Subtheme: Reduced importance of oral health and oral care***	
B7	*I simply cannot brush my teeth properly anymore. […] But I don’t mind having to take dentures. […] My health is more important than my teeth now. (man, 80, severe Parkinson, severely frail).*
B8	*When you can’t do anything anymore, then you don’t wish to do anything anymore, then you can’t be bothered about anything. (woman, 85, severely frail).*
B9	*My teeth don’t interest me. Because I am depressed. […] I only rinse them [dentures] when something gets underneath, and that’s it. […] I don’t know if a dentist could help me, I don’t care. (woman, 73, moderately frail).*
B10	*I can’t get them 100% clean, not even with an electric toothbrush […] It is too hard to reach them […] I’ve tried, but it didn’t work, and now it doesn’t bother me anymore. […] I don’t mind losing my teeth. (man, 80, severely frail).*
B11	*I wouldn’t [see a dentist], not unless I would have serious toothache. Life won’t last that long anymore when you’re so old as I am. […] My teeth will keep, I think. (woman, 85, severely frail).*
B12	*I’m only bothered with having a fresh feel in my mouth now […] when you’ve kept your teeth this long like me, they will survive. (woman, 84, moderately frail).*
B13	*I would not go to the dentist [in order to replace bad teeth]. […]. If I cannot bite anymore I will eat porridge. (woman, 93, severely frail).*
***Subtheme: Conscious choice to preserve energy for other goals***	
B14	*I don’t see a dentist anymore. I don’t feel like it. I rather preserve my energy for other things. […] But if I would have pain, I would go again. I wouldn’t go on with a painful mouth. (woman, 77, severe arthritis, severely frail).*
B15	*When I can achieve, with only a small effort, that my mouth remains fresh and a bit healthy, then I don’t mind doing it, but if it takes a big effort, then not, which is why I don’t see a dentist anymore. (woman, 93, severely frail).*
B16	*And in the past I would clean my dentures after a meal, but, and that is laziness, I openly admit it, I don’t do that anymore. […] After all it takes an effort, and I have to divide my energy sensibly. I could go walk back and forth to the bathroom, but I rather be knitting something, or do something else. (woman, 86, severely frail).*
B17	*I don’t wish to look for another dentist, because that requires a lot of you. When you get older and weaker […] you can’t work up the effort. I could do it when I was younger, but now, look I don’t cycle anymore. I am just slower […]. It really is not important enough. […] And now I need to look after my husband [*a Parkinson patient*], and I have to save all my time and effort for that. (woman, 80, slightly frail).*

**Table 5:** Theme C and supporting quotations

***Theme C: Structural barriers: I’d like to, but I can’t***	
***Subtheme: Disorientation: I don’t know how it works here***	
C1	*Since I live here, I don't always get the right care. Because I don't know how it works when I need care here, if I should go back to see my old dentist or if they [staff] arrange someone. I wouldn't know. (woman, 86, recently institutionalized, slightly frail).*
C2	*I wouldn't mind seeing a dentist, but I don't know anyone here. I don't know who would be good. […] Everything is so distressing here. (woman, 79, moderately frail).*
C3	*I would have to look up where to go to. I am not at home anymore. And I don't have all the addresses anymore. So to find all that out, that is an enormous....But I should do it. I should look up where my own dentist is. And then i should go. It has been too long ago since I went there. (woman, 93, severely frail).*
C4	*I have to brush regularly. And, you should write this down, that does not happen here. They forget to help remind me. You have to do it yourself […] and then I lie on my bed and I think, oh my God, I did not brush my teeth. And I cannot walk by myself, I need someone to bring me to the bathroom […] They don’t help me enough. I am forgetful now, and they don’t remind me.[…] I have looked after my teeth my whole life, and now they let it get in a mess. (woman, 93, severely frail).*
C5	*It is a bit difficult with my hands […] and to reach the wash basin. […] [interviewer: why haven’t you asked the nurses to help you?]. I didn’t think about it, didn’t know I could do that. (man, 65, spastic, wheel chaired, severely frail).*
***Subtheme: Inconveniencing social support: getting (the right) help is hard.***	
C6	*I do want to have it fixed. […] but I cannot burden my daughter to take me to the dentist as well. She has had enough on her plate. [interviewer: and have you considered asking your other children?] Well I have asked it enough. I cannot go on insisting. “Mum, stop nagging,” they say to me. (woman, 83, severely frail).*
C7	*I would only go now if I would have pain. And then I would ask my daughter to bring me to the dentist. […] I would only go if she can make it, because she’s busy herself. (woman, 97, severely frail).*
C8	*I still live independently and I have to bother people with my requests so often, and I have to ask so many people to do something for me, and I don’t like that. (woman 80, slightly frail).*
C9	*The whole inside hurts because of my lower dentures. And I thought, I should go the dentist, but well, I don’t have a husband no longer, and that means I would have to go there myself. […] So I haven’t gone yet. (woman, 87, slightly frail).*
C10	*I think that a nurse does not like to brush my teeth. A nurse is not really paid to do it, has not been trained to do it […] that makes it hard to accept help, the thought that people do not like to help you brush, it makes you feel so dependent. (woman, 80, slightly frail).*

### Theme A: oral hygiene routines sustain a sense of self worth

There was a strong desire to remain the same person as before the onset of health decline, if not through maintaining the same level of oral health, then at least through adherence to the same daily oral hygiene routines. The importance of adhering to routines seemed even stronger for people who felt quite weak; it helped them to sustain their sense of autonomy and self-control, and hence self-worth. Some severely frail participants continued to brush their teeth daily, despite physical difficulties (Table 3, qA1), in an attempt, especially among ‘younger’ (65 – 80 y.o.) women, to appear well-groomed.

One man in an intensive care unit explained how he brushed his teeth in order to feel ‘normal’ as soon as he was well enough to get out of bed (qA2). This also applied to severely frail people who needed help with their daily oral routines, and who wanted to maintain their dignity by being and feeling well cared for (qA3). Support and attention from staff was not only expected to increase oral hygiene; it also made people feel worthy of care “*I still count*” (qA4). Those who were less dependent felt that mouth-care demonstrated self-control (qA5), and they associated neglect of their mouth with human decay and loss of dignity. Only a minority seemed unconcerned about discontinuing oral hygiene routines and losing control associated with oral self-care. These were mostly males, edentates, people who had never cared much about their oral health and a few severely frail people distressed by pain.

Maintenance of formerly established oral hygiene-related behavior contributed to self-worth not only through the concept of self in relation to “I” (how I see myself) (qA2 – A4, A6), but also through the concept of self in relation to others (how others see me) (qA7, A8). In the latter case, the contribution of oral hygiene-related behavior to self-worth was related to the extent of social involvement of people and the extent to which they valued this social involvement. People who enjoyed frequent visits from friends or relatives or who actively participated in social activities, generally put more emphasis on the social aspect of a clean mouth, than did those who were less socially active.

For only two people did use of dental services also contribute to their perception of themselves as normal functioning human beings, albeit to a lesser extent than toothbrushing did. With increasing frailty people abandoned their dental visits much sooner than their daily toothbrushing routines.

### Theme B: lack of motivation: the benefits of dental visits or daily tooth cleaning are not worth the effort

The majority of wearers of complete (full) and removable partial dentures and the majority of the frailest, institutionalized participants, did not see a dentist anymore. Most said that they did not feel they needed to go, or that it required too much effort with no obvious benefit, which was remarkable since about half of the participants who did not see a dentist anymore complained about uncomfortable and loose dentures, loose teeth or painful spots. A small minority of people, all severely frail with impaired mobility or dexterity or with low energy, also reduced their toothbrushing frequency or stopped cleaning their teeth altogether.

We identified three underlying subthemes that explained reduced motivation.

### Lack of belief in results

Most complete denture-wearers had stopped making dental visits, either because of bad experiences with dentists and denture-makers, or because they had not been to a dentist for many (often 20 – 30) years and could not imagine how a dentist could help them (Table 4, qB1,B2). The general conviction among denture-wearers was that dentures are unavoidably uncomfortable, and complicated by old age, diseases or even poor genes (qB3, B4), and that relief was more easily obtained by simply not wearing the lower denture than by visiting a dentist. A minority of dentates had stopped making dental visits (qB5) mostly because they did not perceive any benefits of these visits other than pain relief.

The perception that dentists are unhelpful might also have been a cover for the belief that visiting a dentist needed too much effort, which some felt was shameful to admit.

With respect to tooth cleaning, a few participants mentioned that they lacked motivation because they did not believe that they could effectively clean their teeth (qB6, B9, B10). This lack of self efficacy was a result of physical impairments and was confirmed by unsatisfying results of cleaning efforts.

### Reduced importance of oral health and oral care

Awareness of declining health, especially in the very old, had two effects on attitude towards oral health. Interest in preventing oral disease was lost as frailty increased (qB7). Participants with low morale or chronic pain or severe impairments that absorbed their vitality, lost interest in oral care (qB7-B9). Others with poor dexterity resulting from Parkinson’s disease, rheumatoid arthritis or other disabling disorders, trivialized oral health when they realized that they could not clean their teeth effectively (qB9, B10).

Health decline in old age also had another effect on attitudes towards oral health and oral health behaviour: People realized that since death was close, the teeth that they had would probably last without professional care or major discomfort (qB11 - B13). Thus, even if dentists were willing to make home-visits, some participants said that they would refuse professional care unless the mouth or tooth pain would become unbearable.

### Conscious choice to preserve energy for other goals

When people indicated that they did not brush their teeth as often as before or had stopped seeing a dentist, the underlying reason was often a conscious decision to use their scarce energy in other ways. The presumed investment of energy into dental visits, a higher brushing frequency, or flossing, did not weigh up against the perceived benefits, unless the perceived benefit was relief of serious pain or discomfort (qB14-B16).

Severely frail people with low energy levels due to mental or physical impairments, were well aware that they had to spread their energy over actions that they considered important or worthwhile. While for most, daily tooth brushing was still important enough to do, seeing a dentist was not (qB14) or required an amount of effort that could be better spent in other ways (qB17), a view that was even shared by some slightly frail people. For most participants the perceived benefits of tooth brushing (mainly, having fresh breath and feeling clean and well-groomed) outweighed the negative consequences of having to make the effort, or remind a nurse to do it (qB15). However, this balance seemed to go in the opposite direction for a few severely frail participants, some of them bedridden, who chose to diminish the frequency of their oral hygiene routines (qB16).

### Theme C: Structural barriers: I’d like to, but I can’t

Besides the people who lacked motivation to see a dentist or maintain their old toothbrushing behavior, there were also people who encountered external barriers to dental visits or oral hygiene practices as a result of frailty-related limitations. The main factors identified as direct disablers of oral care behavior, were diminished mobility and dexterity, disorientation, failing memory and dependence on, or lack of support from others, all of which have been documented before. However, it was noticed that, in contrast to psychological and social barriers, physical barriers, like being wheelchair-bound were often not in themselves sufficient motivators for giving up or altering oral care behavior. Rather, these barriers accumulated with other factors and then made the balance of required efforts versus perceived benefits tip over to the ‘too much effort’ side, especially in severely frail people.

It was noticed that in particular the effect of psychological and social factors seemed to be reinforced by institutionalization, and two related themes emerged.

### Disorientation: I don’t know how it works here

Being institutionalized constituted a major change in oral health behavior for many severely and moderately frail people. After arriving at a home, people often stopped seeing a dentist. This was either because their old dentist was too far away, or because they had not been informed if the home had its own dentist or not and hence did not know if they should keep seeing their old dentist or not, or because they had been informed about the home’s dentist but not about how to arrange dental visits (Table 5, qC1). A lot of people, even after two or more years, were still getting used to the new environment and routines in their care homes. Assessing the dental care situation, let alone organizing a visit, did not have their attention or had low priority. There was a plain element of distress in most accounts (qC2), because people thought that they ought to see a dentist but felt that they were not up to the task of either arranging a visit or of getting there (qC3).

Disorientation, albeit to a lesser extent and mostly in people who were mentally frail, also played a role in daily hygiene routines of institutionalized participants (qC4).

Some disabled people had reluctantly given up toothbrushing because they could not do it themselves and had not considered asking help from a nurse, because they had ‘*never thought about it’* and were clearly unaware of the possibility of getting assistance from staff (qC5).

### Inconveniencing social support: getting (the right) help is hard

Although most people were aware that they could ask for help to arrange and make dental visits, and although almost everyone could name someone that they could ask for help, they were very careful not to overburden their relations (qC6). In most cases there was a long list of actions that required help from others, and making a dental visit was often not among the most urgent ones. For most people, the only reason that justified asking for help from others, was oral pain (qC7). Barriers related to social support also played a role for a few non-institutionalized people who lived alone (qC8, C9).

Complaints about the support they received from nurses were not limited to reminders to brush or clean dentures (qC4). Nurses, it was said, did not put the brush or the dentures back in the same place every time, they were too rushed, and did not always clean or rinse dentures properly, so that they remained dirty or tasted of soap.

Almost all participants wished to keep their independence and insisted on brushing their teeth themselves for as long as their general health allowed them to do so. People with disabling disorders like impaired dexterity or vision, incessantly had to weigh up their need for properly brushed teeth against their loss of independence. The thought of losing independence was clearly mitigated by the attitude of the caregiver, who, according to several participants, could make the difference between people’s asking for help and accepting it or people neglecting their oral care (qC10).

## Discussion

### New insights and possible explanations

This is the first study to our knowledge that provides in-depth insight into the pathways through which manifestations of frailty affect oral care behavior, particularly with regard to continuation or cessation of oral care behavior. We identified several established frailty factors [34] that influenced oral care behavior in different ways: chronic pain, impaired mobility, impaired dexterity, low energy (physical frailty), disorientation, bad memory, low morale (psychological frailty), and lack of support (social frailty). Chronic pain, low energy and low morale mainly affected oral care behavior through devaluation of oral health importance (attitudes) and by reducing motivation. Physical constraints reduced self efficacy beliefs with regard to oral hygiene practices, while bad past experiences, often in combination with reduced motivation, affected outcome expectations with regard to dental visits, especially for denture wearers. Impaired mobility and dexterity, disorientation, failing memory and lack of social support constituted structural barriers to oral care behavior that could only be reduced by others, and institutionalization seemed to increase the effects of psychological and social frailty factors on oral care behavior.

Identified frailty factors, often in combination with a lack of belief that a dentist could improve their oral health, together with increasing frailty and/or institutionalization caused most people to decrease or end their dental service use, but not abandon daily hygiene routines. This was because toothbrushing, in contrast to dental check-up visits, was seen as a necessary and manageable effort for maintaining good oral health, and because adhering to formerly established toothbrushing routines helped frail people feel ‘normal’ and hence maintain self-worth and dignity.

While the role of self-worth, in particular autonomy, in adherence to general health care routines for institutionalized elderly has been documented [35,36], as has the role of self-worth in having natural teeth in old age [37], no literature, to our knowledge, explicitly links self-worth and oral hygiene-related behavior for this group.

Character traits, particularly psychosocial constructs like self-efficacy [38], locus of control [39], optimism [40], sense of coherence [41], hostility [42], coping and adaptation [43], and resilience [44], have a proven influence on oral hygiene-related behavior. Our interviews seemed to support the already large body of evidence implying that self-efficacy has a vital influence on oral care behavior [18,19,38,45-49]. We also found some support for the view that people with a high internal locus of control (interpreting events as being dependent on his/her own behavior) would less readily give up their dental check-up visits and toothbrushing than people with external locus of control, while people who seemed good at adapting to their impaired health would give up dental check-up visits easily or not mind if they could not clean their teeth properly.

This study shows that commonly recognized barriers to dental service-use by elderly, ‘availability’, ‘accessibility’, ‘cost’, ‘dependence on others’ and, in some cases, even ‘perceived oral problems’ seem to be of only secondary importance in the studied group. When we mentioned the possibility of free dental check-ups through use of mobile dental units, most people who had stopped seeing a dentist were not convinced that they would use them or plainly stated that they would not go, thus providing evidence against the statement ‘if you built it, they will come.’ (see [13]). This was the more remarkable since the majority of this group admitted that they experienced some degree of oral discomfort. The majority of severely frail people simply did not wish to see a dentist because the perceived benefits were small or non-existent and did not outweigh the perceived required efforts, even if the required efforts would be minimized through provision of dental check-ups at home.

The motives for both continuation and discontinuation of oral care can be understood with help of the Motivational Theory of Life Span Development [50], in particular the Goal Engagement and Goal Disengagement control strategies [51] which form part of this theory. The theory proposes that the key criterion for adaptive development is the extent to which someone realizes control of his or her environment across different domains of life and across the life span. Vital to this theory is the assumption that people try to optimize control over their lives and adjust goals and strategies to achieve this according to their circumstances. According to the circumstances, someone will either use primary control strategies (directed at changing the environment in order to bring it in line with one’s wishes) or secondary control strategies (directed at changing the self to bring it in line with the environment). Secondary control strategies are used when primary control strategies are not available or fail and comprise (a) adjustment of goals or standards and engaging in self-protective attributions and favorable comparisons (selective secondary control), and (b), in case a goal becomes unattainable, goal disengagement, and freeing up resources (time, effort, motivation, skills) for the pursuit of more attainable goals, sometimes in different domains of life (compensatory secondary control) [50,52].

To most of our participants, the goal of a fresh and clean mouth remained attainable through the practice of tooth cleaning, which rendered a feeling of control that may be seen as a goal in itself (‘I can still manage’).

With increasing frailty, people compared oral discomfort to other, more troubling, health problems, or attributed it to old age or genetic factors They judged their oral health by comparing it to what they perceived as normal for their age or health situation, and not to a completely healthy mouth (selective secondary control). Many people thought it was normal to have ill-fitting dentures, because they heard so many people complain about them. Hence their norm for ‘good oral health’ differs from the clinician’s norm. This helps explain the discrepancy between normative and perceived treatment need [2,53].

With increasing frailty, people tended to judge that the perceived effort required for seeing a dentist, or, in some cases, brushing their teeth efficiently, did not weigh up against the perceived benefits. They consequently disengaged from the goal (optimal oral health) that motivated these practices. Using compensatory secondary control strategies, they devalued the goal (‘oral health is not so important anymore’), lowered the outcome expectation of the behavior (‘the dentist cannot help me anyway’, or: ‘even when I brush, my teeth don’t get clean’), adapted to minor oral discomfort, and consciously preserved their motivational resources for more attainable and rewarding goals (‘I’d rather use my energy for knitting’), thus providing more insight into the discrepancy between treatment need and service use [1-6].

Contrary to earlier statements implying that frail people discontinue oral care behavior because their impairments render them apathetic [31] (p.200), our findings, in the light of the Motivational Theory of Life Span Development, suggest that this discontinuation by many frail elderly may be interpreted as an expression of adaptive development. A model guided study is needed to further investigate these assumptions.

In terms of Anderson’s and Newman expanded model [54,55], which was originally aimed at predicting health services usage from three dynamics: predisposing, enabling and need factors, our results suggest that predisposing factors, especially health attitudes (the importance attributed to oral health) and beliefs (the difference that a dentist or toothbrushing session can make to general well being, the severity of perceived health risk, and self efficacy) are likely to play a more important role in predicting the oral health behavior of frail elderly, than do need factors, except in case of pain.

This finding is supported by earlier evidence regarding dental service-use by the elderly [4] and toothbrushing by adults [19] and by many studies on the impact of self efficacy on oral care behavior [18,19,38,45-49].

### Methodological strength and limitations

Our study design enabled comparison of perspectives of people with different degrees and characteristics of frailty. Severe cognitive disorders have been shown to have a major impact on oral care behavior as well, but our methodology (interviews) did not allow inclusion of cognitively impaired participants, who generally have worse oral health status and face more barriers to constructive oral health behavior than the majority of our participants [56,57].

As selection of participants was based on voluntarily participation after being informed by the care-manager, it is likely that the number of participants with low socioeconomic status (SES) and with less favorable health behavior and health attitudes was relatively low. These factors are known to reduce willingness to participate in research projects [58]. As a result of this selection bias, themes identified in this study are likely to predominantly represent the views of older people with relatively high SES and relatively favorable oral health attitudes. Based on the expected low number of participants with low SES and on the exclusion of cognitively impaired people, it can be assumed that oral care behavior among frail Dutch older people is less favorable and perhaps even more affected by physical, cognitive and social impairments than our study suggests.

Although SES, cognitive status and also cultural background are factors that have a proven influence on oral health care in general [56,59-61], we chose to focus on various manifestations and degrees of frailty and to limit the number of varying dimensions to those that we expected to be of major influence, in order to warrant analytical strength. For the same reason, we did not study the effect of character traits.

Interviews were conducted by two researchers who had no background in medicine, geriatrics or dentistry. This probably helped to make participants feel free to inform the interviewers about their ‘poor’ oral health behavior or unfavorable oral health attitude. However, this also entailed the restriction that self reported health and oral health issues and experiences with dentists could not always be interpreted against a relevant clinical background during the interview. Regular consultation with the third researcher, a dentist, and with a geriatric dentist helped to overcome this shortcoming. Likewise, we consulted the care-managers to check unclear, implausible, or contradictory information from the participants in order to reduce information bias caused by social desirability.

### Implications for dental care professionals and nursing staff

In attempting to improve the oral care behavior of frail elderly, it may be useful to distinguish between factors that prevent people from applying oral care regardless of their wishes (like reduced mobility and dexterity, disorientation, failing memory, and lack of support), and factors that make people unwilling to continue applying oral care any longer (like chronic pain, low energy, low morale). The first type of factors can be addressed through early signaling of problems and provision of adequate oral hygiene support by nurses. In dealing with the second type of factors, nurses and dental professionals need to first weigh up clinical and oral hygiene related benefits of interventions against the autonomy of the patient.

Our study contributes to the discussion about the nature and frequency of required professional oral care for this group, and about the allocation of resources, that can be justified either by patient-outcomes or by clinical outcomes.

It can be argued that, especially for a population of frail elderly people who are generally more concerned about short term than long term health benefits, clinical outcomes are less meaningful to patients than patient based outcomes, like discomfort and quality of life. More generally, in evaluations of the efficacy of health services, the perspective of the patient is becoming increasingly important and in some cases even replaces the perspective of the clinicians [62]. Our study results show that, from a patient perspective, resources can be better allocated to support with daily oral hygiene than to dental service-use, unless the service is used for relief of perceived pain or discomfort. Perceived health benefits of oral care, besides pain relief, are mainly social and psychological in nature: Functionally impaired elderly people who get help with their oral hygiene perceive that they are still worthy of being cared for. This, in addition to looking and being well-groomed, enhances their sense of self-worth and social worth. This directly improves their quality of life, whereas the perceived health benefits of preventive or even restorative dental visits are not always obvious. Such benefits could not be established in a longitudinal study by Locker [63].

In recent years, provision of dental care through mobile units has become an increasingly widespread practice in Northern European countries, the USA, Canada and Australia [64-69]. It can indeed solve problems for those with oral pain or discomfort who are unable to attend regular dental practices due to lack of transport or mobility problems. However, providing mobile dental care to frail older people regardless of their treatment demands and regardless of their abilities to arrange and make dental visits by themselves, is likely to be cost-ineffective and is also at variance with people’s autonomy rights. The primary aim of dental care should be to keep severely frail people free of oral pain and discomfort. For most residents who were interviewed in this and a previous study by the same authors [37], this could be achieved by nurses or carers providing necessary support in daily hygiene routines, and through arranging dental visits and transport in cases requiring treatment.

Measures that target the interaction between residents and nursing staff and that increase the quality and level of care without any substantial cost, could relieve most of the barriers to favorable oral care behavior that we observed in this study. Compassionate care and patient centered communication, for instance, are two related approaches that have been proven to enhance the quality of care in care dependent older people [70-74]. They include close observation of patients and effective and empathic communication, and lead to reduction of medical errors and improved health outcomes and patient satisfaction [71]. These approaches are expected to reduce barriers to oral health care encountered in this and other studies [69,75,76], including the invisibility and underreporting of the resident’s oral health concerns. Close observation of residents and empathic communication could be used to learn about and understand the resident’s concerns and wishes, his or her health priorities, oral health attitude and experienced barriers to good oral hygiene practices. Dental and nursing staff should also be alert to indicators for poor (oral) hygiene-related behavior, like forgetfulness, depression, or poor dexterity. More specifically, nurses and dentists should regularly ask residents if they experience difficulties in tooth brushing or organizing a dental visit. Compassionate care will help improve the relationship between dentist and patient and between nurse and resident, and may increase the nurse’s willingness to support residents with their oral care. As a result, two of the most frequently reported barriers to oral health care support by nursing staff, lack of prioritization and unfavorable oral healthcare attitude [69,75,77-79], may be mitigated.

While several studies have evaluated the effects of training programs for nurses and care-aides aimed at improving oral care support, e.g. [79-82], or have documented barriers to oral care provision for this group, e.g.[83-88], one major barrier to accurate oral care support from nurses remains largely unaddressed. Good oral health of residents is generally not incorporated in the list of performance indicators that serve to evaluate the quality of a residence and its managers. As long as managers cannot be held responsible for deficient oral health management, implementation of any training program or oral health care guidelines for institutionalized elderly is likely to be ineffective in the long term.

Empowering the patient to express his or her oral hygiene needs may help. Empowering the patient is at least not subject to the usual high staff turnover, time and money constraints and lack of management support that undermine the effectiveness of training programs for health carers [89].

## Competing interests

The authors declare that they have no competing interests.

## Authors’ contributions

Dominique Niesten designed the study, conducted interviews, transcribed and analyzed the data and wrote the paper. Krista van Mourik conducted interviews, transcribed and analyzed the data and contributed to the paper. Wil van der Sanden analyzed the data and contributed to the paper. All authors have read and approved the final version of the manuscript.

## Pre-publication history

The pre-publication history for this paper can be accessed here:

http://www.biomedcentral.com/1472-6831/13/61/prepub

## References

[B1] Sanders AE Slade GD Lim S Reisine ST Impact of oral disease on quality of life in the US and Australian populations Community Dent Oral Epidemiol 2009 37 2 171 181 10.1111/j.1600-0528.2008.00457.x 19175659 PMC3760707

[B2] Slade GD Sanders AE The paradox of better subjective oral health in older age J Dent Res 2011 90 11 1279 1285 10.1177/0022034511421931 21917599

[B3] Dahl KE Wang NJ Skau I Ohrn K Oral health-related quality of life and associated factors in Norwegian adults Acta Odontol Scand 2011 69 4 208 214 10.3109/00016357.2010.549502 21247228

[B4] Kiyak HA An explanatory model of older persons' use of dental services. Implications for health policy Medical care 1987 25 10 936 952 10.1097/00005650-198710000-00003 3695632

[B5] Branch L Jette A Evashwick C Polansky M Rowe G Diehr P Toward understanding elders' health service utilization J Community Health 1981 7 2 80 92 10.1007/BF01323227 7328199

[B6] Grabowski M Bertram U Oral health status and need of dental treatment in the elderly Danish population Community Dent Oral Epidemiol 1975 3 3 108 114 10.1111/j.1600-0528.1975.tb00290.x 1056814

[B7] Branch LG Antczak AA Stason WB Toward understanding the use of dental services by the elderly Spec Care Dentist 1986 6 1 38 41 10.1111/j.1754-4505.1986.tb00949.x 3515592

[B8] Gerritsen PF Cune MS van der Bilt A de Putter C Dental treatment needs in Dutch nursing homes offering integrated dental care Spec Care Dentist 2011 31 3 95 101 10.1111/j.1754-4505.2011.00185.x 21592163

[B9] Ferro R Besostri A Strohmenger L Mazzucchelli L Paoletti G Senna A Stellini E Mazzoleni S Oral health problems and needs in nursing home residents in Northern Italy Community Dent Health 2008 25 4 231 236 19149301

[B10] Gobbens RJ Luijkx KG Wijnen-Sponselee MT Schols JM In search of an integral conceptual definition of frailty: opinions of experts J Am Med Dir Assoc 2010 11 5 338 343 10.1016/j.jamda.2009.09.015 20511101

[B11] MacEntee MI Oral healthcare and the frail elder 2011 Iowa: Wiley-Blackwell

[B12] Borreani E Wright D Scambler S Gallagher JE Minimising barriers to dental care in older people BMC Oral Health 2008 8 7 10.1186/1472-6831-8-7 18366785 PMC2335092

[B13] Kiyak HA Reichmuth M Barriers to and enablers of older adults' use of dental services J Dent Educ 2005 69 9 975 986 16141083

[B14] Dolan TA Atchison KA Implications of access, utilization and need for oral health care by the non-institutionalized and institutionalized elderly on the dental delivery system J Dent Educ 1993 57 12 876 887 8263235

[B15] Brothwell DJ Jay M Schonwetter DJ Dental service utilization by independently dwelling older adults in Manitoba, Canada J Can Dent Assoc 2008 74 2 161 161f 18353200

[B16] Dolan TA Peek CW Stuck AE Beck JC Functional health and dental service use among older adults The journals of gerontology Series A, Biological sciences and medical sciences 1998 53 6 M413 418 10.1093/gerona/53a.6.m413 9823744

[B17] Evashwick C Rowe G Diehr P Branch L Factors explaining the use of health care services by the elderly Health Serv Res 1984 19 3 357 382 6746297 PMC1068819

[B18] Buglar ME White KM Robinson NG The role of self-efficacy in dental patients' brushing and flossing: testing an extended health belief model Patient Educ Couns 2010 78 2 269 272 10.1016/j.pec.2009.06.014 19640670

[B19] Anagnostopoulos F Buchanan H Frousiounioti S Niakas D Potamianos G Self-efficacy and oral hygiene beliefs about toothbrushing in dental patients: a model-guided study Behavioral medicine (Washington, DC) 2011 37 4 132 139 10.1080/08964289.2011.636770 22168330

[B20] Jablonski RA Munro CL Grap MJ Elswick RK The role of biobehavioral, environmental, and social forces on oral health disparities in frail and functionally dependent nursing home elders Biol Res Nurs 2005 7 1 75 82 10.1177/1099800405275726 15920005

[B21] Stromberg E Hagman-Gustafsson ML Holmen A Wardh I Gabre P Oral status, oral hygiene habits and caries risk factors in home-dwelling elderly dependent on moderate or substantial supportive care for daily living Community Dent Oral Epidemiol 2012 40 3 221 229 10.1111/j.1600-0528.2011.00653.x 22070521

[B22] Kuthy RA Strayer MS Caswell RJ Determinants of dental user groups among an elderly, low-income population Health Serv Res 1996 30 6 809 825 8591931 PMC1070094

[B23] MacEntee MI Weiss R Waxler-Morrison NE Morrison BJ Factors influencing oral health in long term care facilities Community Dent Oral Epidemiol 1987 15 6 314 316 10.1111/j.1600-0528.1987.tb01742.x 3121247

[B24] Pope C Mays N Qualitative research in health care 2006 3 Oxford: Blackwell publishing

[B25] Patton MQ Qualitative research and evaluationmethods 2002 3 Thousand Oaks, CA: SAGE Publications

[B26] Macek MD Cohen LA Reid BC Manski RJ Dental visits among older U.S. adults, 1999: the roles of dentition status and cost J Am Dent Assoc 2004 135 8 1154 1162 quiz 1165 15387055 10.14219/jada.archive.2004.0375

[B27] Kiyak HA Age and culture: influences on oral health behaviour Int Dent J 1993 43 1 9 16 8478130

[B28] Lester V Ashley FP Gibbons DE Reported dental attendance and perceived barriers to care in frail and functionally dependent older adults Br Dent J 1998 184 6 285 289 10.1038/sj.bdj.4809604 9581365

[B29] MacEntee MI Stolar E Glick N Influence of age and gender on oral health and related behaviour in an independent elderly population Community Dent Oral Epidemiol 1993 21 4 234 239 10.1111/j.1600-0528.1993.tb00763.x 8370262

[B30] Avlund K Holm-Pedersen P Schroll M Functional ability and oral health among older people: a longitudinal study from age 75 to 80 J Am Geriatr Soc 2001 49 7 954 962 10.1046/j.1532-5415.2001.49187.x 11527488

[B31] Frenkel H Matthews DC Nitschke I MacEntee CI Prevention of oral diseases for a dependent population Oral Health Care and the Frail Elder A Clinical Perspective 2011 Iowa: Wiley-Blackwell 187 210

[B32] Krefting L Rigor in qualitative research: the assessment of trustworthiness Am J Occup Ther 1991 1991 45 214 222 2031523 10.5014/ajot.45.3.214

[B33] Strauss A Corbin J Basics of Qualitative Research Techniques and Procedures for Developing Grounded Theory 1998 2edn London: Sage Publications

[B34] Gobbens RJ van Assen MA Luijkx KG Schols JM Testing an integral conceptual model of frailty J Adv Nurs 2012 68 9 2047 2060 10.1111/j.1365-2648.2011.05896.x 22150238

[B35] Sacco-Peterson M Borell L Struggles for autonomy in self-care: the impact of the physical and socio-cultural environment in a long-term care setting Scand J Caring Sci 2004 18 4 376 386 10.1111/j.1471-6712.2004.00292.x 15598245

[B36] Blair CE Effect of self-care ADLs on self-esteem of intact nursing home residents Issues Ment Health Nurs 1999 20 6 559 570 10.1080/016128499248367 10839045

[B37] Niesten D van Mourik K van der Sanden W The impact of having natural teeth on the QoL of frail dentulous older people. A qualitative study BMC public health 2012 12 1 839 10.1186/1471-2458-12-839 23031489 PMC3524040

[B38] Syrjala AM Kneckt MC Knuuttila ML Dental self-efficacy as a determinant to oral health behaviour, oral hygiene and HbA1c level among diabetic patients J Clin Periodontol 1999 26 9 616 621 10.1034/j.1600-051X.1999.260909.x 10487313

[B39] Kneckt MC Syrjala AM Knuuttila ML Locus of control beliefs predicting oral and diabetes health behavior and health status Acta Odontol Scand 1999 57 3 127 131 10.1080/000163599428841 10480277

[B40] Ylostalo P Ek E Knuuttila M Coping and optimism in relation to dental health behaviour–a study among Finnish young adults Eur J Oral Sci 2003 111 6 477 482 10.1111/j.0909-8836.2003.00083.x 14632683

[B41] Savolainen JJ Suominen-Taipale AL Uutela AK Martelin TP Niskanen MC Knuuttila ML Sense of coherence as a determinant of toothbrushing frequency and level of oral hygiene J Periodontol 2005 76 6 1006 1012 10.1902/jop.2005.76.6.1006 15948698

[B42] Mettovaara HL Suominen-Taipale AL Uutela AK Martelin TP Knuuttila ML Cynical hostility as a determinant of toothbrushing frequency and oral hygiene J Clin Periodontol 2006 33 1 21 28 10.1111/j.1600-051X.2005.00864.x 16367851

[B43] MacEntee MI Hole R Stolar E The significance of the mouth in old age Soc Sci Med 1997 45 9 1449 1458 10.1016/S0277-9536(97)00077-4 9351161

[B44] Martins AB Dos Santos CM Hilgert JB de Marchi RJ Hugo FN Pereira Padilha DM Resilience and self-perceived oral health: a hierarchical approach J Am Geriatr Soc 2011 59 4 725 731 10.1111/j.1532-5415.2011.03350.x 21438867

[B45] Kakudate N Morita M Fukuhara S Sugai M Nagayama M Kawanami M Chiba I Application of self-efficacy theory in dental clinical practice Oral Dis 2010 16 8 747 752 10.1111/j.1601-0825.2010.01703.x 20646233

[B46] Syrjala AM Ylostalo P Niskanen MC Knuuttila ML Relation of different measures of psychological characteristics to oral health habits, diabetes adherence and related clinical variables among diabetic patients Eur J Oral Sci 2004 112 2 109 114 10.1111/j.1600-0722.2004.00113.x 15056106

[B47] Callaghan D Healthy behaviors, self-efficacy, self-care, and basic conditioning factors in older adults J Community Health Nurs 2005 22 3 169 178 10.1207/s15327655jchn2203_4 16083404

[B48] Tedesco LA Keffer MA Fleck-Kandath C Self-efficacy, reasoned action, and oral health behavior reports: a social cognitive approach to compliance J Behav Med 1991 14 4 341 355 10.1007/BF00845111 1942013

[B49] Stewart JE Strack S Graves P Development of oral hygiene self-efficacy and outcome expectancy questionnaires Community Dent Oral Epidemiol 1997 25 5 337 342 10.1111/j.1600-0528.1997.tb00951.x 9355768

[B50] Heckhausen J Wrosch C Schulz R A motivational theory of life-span development Psychol Rev 2010 117 1 32 60 20063963 10.1037/a0017668 PMC2820305

[B51] Heckhausen J Staudinger UM, Lindenberger U The future of lifespan psychology: Perspectives from control theory Understanding human development: Dialogues with lifespan psychology 2003 Dordrecht, the Netherlands: Kluwer Academic 383 400

[B52] Ebner NC Freund AM Blackburn JA, Dulmus CN Personality Theories of Successful Aging Handbook of Gerontology Evidence Based Approaches to Theory, Practice and Policy 2007 New Jersey: John Wiley and Sons 87 116

[B53] Locker D Gibson B Discrepancies between self-ratings of and satisfaction with oral health in two older adult populations Community Dent Oral Epidemiol 2005 33 4 280 288 10.1111/j.1600-0528.2005.00209.x 16008635

[B54] Andersen R Newman JF Societal and individual determinants of medical care utilization in the United States Milbank Mem Fund Q Health Soc 1973 51 1 95 124 10.2307/3349613 4198894

[B55] Andersen RM Revisiting the behavioral model and access to medical care: does it matter? J Health Soc Behav 1995 36 1 1 10 10.2307/2137284 7738325

[B56] Wu B Plassman BL Liang J Wei L Cognitive function and dental care utilization among community-dwelling older adults Am J Public Health 2007 97 12 2216 2221 10.2105/AJPH.2007.109934 17971546 PMC2089110

[B57] Jablonski RA Kolanowski A Therrien B Mahoney EK Kassab C Leslie DL Reducing care-resistant behaviors during oral hygiene in persons with dementia BMC Oral Health 2011 11 30 10.1186/1472-6831-11-30 22100010 PMC3231974

[B58] Lorant V Demarest S Miermans PJ Van Oyen H Survey error in measuring socio-economic risk factors of health status: a comparison of a survey and a census Int J Epidemiol 2007 36 6 1292 1299 10.1093/ije/dym191 17898025

[B59] Tsakos G Demakakos P Breeze E Watt RG Social gradients in oral health in older adults: findings from the English longitudinal survey of aging Am J Public Health 2011 101 10 1892 1899 10.2105/AJPH.2011.300215 21852627 PMC3222342

[B60] McGrath C Bedi R The importance of oral health to older people's quality of life Gerodontology 1999 16 1 59 63 10.1111/j.1741-2358.1999.00059.x 10687509

[B61] Wu B Plassman BL Liang J Remle RC Bai L Crout RJ Differences in self-reported oral health among community-dwelling black, Hispanic, and white elders J Aging Health 2011 23 2 267 288 10.1177/0898264310382135 20858912 PMC3129602

[B62] Hao Y Patient-reported outcomes in support of oncology product labeling claims: regulatory context and challenges Expert Rev Pharmacoecon Outcomes Res 2010 10 4 407 420 10.1586/erp.10.45 20715918

[B63] Locker D Does dental care improve the oral health of older adults? Community Dent Health 2001 18 1 7 15 11421409

[B64] Bee JF Portable dentistry: a part of general dentistry's service mix Gen Dent 2004 52 6 520 526 15636278

[B65] da Fonseca AC C Montenegro FL Assessment of mobile dental services in the State of Lower Austria, Austria Gerodontology 2009 26 4 302 304 10.1111/j.1741-2358.2009.00288.x 19555358

[B66] Milner WE Special care dentistry delivers a formula for change: a model has been developed but must be implemented statewide N C Med J 2005 66 6 460 464 16438104

[B67] Sjogren P Forsell M Johansson O Mobile dental care Br Dent J 2010 208 12 549 550 20577227 10.1038/sj.bdj.2010.546

[B68] Mobile Dentistry Mobile Care for Seniorshttp://www.cmchealthyathome.org/body.cfm?id=17

[B69] Paley GA Slack-Smith L O'Grady M Oral health care issues in aged care facilities in Western Australia: resident and family caregiver views Gerodontology 2009 26 2 97 104 10.1111/j.1741-2358.2008.00230.x 19490132

[B70] Hong J Nguyen TV Prose NS Compassionate care: enhancing physician-patient communication and education in dermatology: part II: patient education J Am Acad Dermatol 2013 68 3 e361 310 364 10.1016/j.jaad.2012.10.060 23394924

[B71] Nguyen TV Hong J Prose NS Compassionate care: enhancing physician-patient communication and education in dermatology: part I: patient-centered communication J Am Acad Dermatol 2013 68 3 e351 358 353 10.1016/j.jaad.2012.10.059 23394923

[B72] Zeber JE Copeland LA Hosek BJ Karnad AB Lawrence VA Sanchez-Reilly SE Cancer rates, medical comorbidities, and treatment modalities in the oldest patients Crit Rev Oncol Hematol 2008 67 3 237 242 10.1016/j.critrevonc.2008.02.002 18356072

[B73] Dechamps A Diolez P Thiaudiere E Tulon A Onifade C Vuong T Helmer C Bourdel-Marchasson I Effects of exercise programs to prevent decline in health-related quality of life in highly deconditioned institutionalized elderly persons: a randomized controlled trial Arch Intern Med 2010 170 2 162 169 10.1001/archinternmed.2009.489 20101011

[B74] Ferrell B Virani R Malloy P Kelly K The preparation of oncology nurses in palliative care Semin Oncol Nurs 2010 26 4 259 265 10.1016/j.soncn.2010.08.001 20971406

[B75] De Visschere L de Baat C De Meyer L van der Putten GJ Peeters B Soderfelt B Vanobbergen J The integration of oral health care into day-to-day care in nursing homes: a qualitative study Gerodontology 2013 2013 2013 (Epub ahead of print). 10.1111/ger.12062 23786637

[B76] Yoon MN Steele CM Health care professionals' perspectives on oral care for long-term care residents: nursing staff, speech-language pathologists and dental hygienists Gerodontology 2012 29 2 e525 535 10.1111/j.1741-2358.2011.00513.x 22462684

[B77] Macentee MI Caring for elderly long-term care patients: oral health-related concerns and issues Dent Clin North Am 2005 49 2 429 443 10.1016/j.cden.2004.10.008 15755414

[B78] Sweeney MP Williams C Kennedy C Macpherson LM Turner S Bagg J Oral health care and status of elderly care home residents in Glasgow Community Dent Health 2007 24 1 37 42 17405469

[B79] Brady M Furlanetto D Hunter RV Lewis S Milne V Staff-led interventions for improving oral hygiene in patients following stroke Cochrane Database Syst Rev 2006 4 CD003864 17054189 10.1002/14651858.CD003864.pub2

[B80] van der Putten GJ De Visschere L Schols J de Baat C Vanobbergen J Supervised versus non-supervised implementation of an oral health care guideline in (residential) care homes: a cluster randomized controlled clinical trial BMC Oral Health 2010 10 17 10.1186/1472-6831-10-17 20598123 PMC2912776

[B81] MacEntee MI Wyatt CC Beattie BL Paterson B Levy-Milne R McCandless L Kazanjian A Provision of mouth-care in long-term care facilities: an educational trial Community Dent Oral Epidemiol 2007 35 1 25 34 10.1111/j.1600-0528.2007.00318.x 17244135

[B82] Nicol R Petrina Sweeney M McHugh S Bagg J Effectiveness of health care worker training on the oral health of elderly residents of nursing homes Community Dent Oral Epidemiol 2005 33 2 115 124 10.1111/j.1600-0528.2004.00212.x 15725174

[B83] Chalmers JM Levy SM Buckwalter KC Ettinger RL Kambhu PP Factors influencing nurses' aides' provision of oral care for nursing facility residents Spec Care Dentist 1996 16 2 71 79 10.1111/j.1754-4505.1996.tb00837.x 9084339

[B84] Chami K Debout C Gavazzi G Hajjar J Bourigault C Lejeune B de Wazieres B Piette F Rothan-Tondeur M Reluctance of caregivers to perform oral care in long-stay elderly patients: the three interlocking gears grounded theory of the impediments J Am Med Dir Assoc 2012 13 1 e1 4 10.1016/j.jamda.2011.06.007 21752721

[B85] Coleman P Improving oral health care for the frail elderly: a review of widespread problems and best practices Geriatric nursing (New York, NY) 2002 23 4 189 199 10.1067/mgn.2002.126964 12183742

[B86] Dharamsi S Jivani K Dean C Wyatt C Oral care for frail elders: knowledge, attitudes, and practices of long-term care staff J Dent Educ 2009 73 5 581 588 19433533

[B87] MacEntee MI Thorne S Kazanjian A Conflicting priorities: oral health in long-term care Spec Care Dentist 1999 19 4 164 172 10.1111/j.1754-4505.1999.tb01380.x 10765882

[B88] de Mello AL Padilha DM Oral health care in private and small long-term care facilities: a qualitative study Gerodontology 2009 26 1 53 57 10.1111/j.1741-2358.2008.00238.x 18510564

[B89] Simons D Baker P Jones B Kidd EA Beighton D An evaluation of an oral health training programme for carers of the elderly in residential homes Br Dent J 2000 188 4 206 210 10740904 10.1038/sj.bdj.4800432

